# An Integrated Genome-Wide Systems Genetics Screen for Breast Cancer Metastasis Susceptibility Genes

**DOI:** 10.1371/journal.pgen.1005989

**Published:** 2016-04-13

**Authors:** Ling Bai, Howard H. Yang, Ying Hu, Anjali Shukla, Ngoc-Han Ha, Anthony Doran, Farhoud Faraji, Natalie Goldberger, Maxwell P. Lee, Thomas Keane, Kent W. Hunter

**Affiliations:** 1 Laboratory of Cancer Biology and Genetics, National Cancer Institute, National Institutes of Health, Bethesda, Maryland, United States of America; 2 Center for Bioinformatics and Information Technology, National Cancer Institute, National Institutes of Health, Bethesda, Maryland, United States of America; 3 Computational Genomics Program, Welcome Trust Sanger Centre, Hinxton, Cambridge, United Kingdom; UCSF Comprehensive Cancer Center, UNITED STATES

## Abstract

Metastasis remains the primary cause of patient morbidity and mortality in solid tumors and is due to the action of a large number of tumor-autonomous and non-autonomous factors. Here we report the results of a genome-wide integrated strategy to identify novel metastasis susceptibility candidate genes and molecular pathways in breast cancer metastasis. This analysis implicates a number of transcriptional regulators and suggests cell-mediated immunity is an important determinant. Moreover, the analysis identified novel or FDA-approved drugs as potentially useful for anti-metastatic therapy. Further explorations implementing this strategy may therefore provide a variety of information for clinical applications in the control and treatment of advanced neoplastic disease.

## Author Summary

Metastasis, the spread and growth of tumor cells from the original tumor to secondary sites throughout the body, is the primary cause of cancer-related death for most solid tumor types. The process of metastasis is very complex, requiring multiple individual steps and the cooperation of different cell types during the dissemination and proliferation steps. Many genes are involved in this process, but at present few have been identified and characterized. In this study, we have integrated multiple genome-wide analysis methods to try to identify large numbers of candidate metastasis-associated genes and pathways based on a highly metastatic mouse model. Using this strategy, we have identified a number of genes that predict outcome of human breast cancer. These genes implicate specific molecular and cellular pathways in the metastatic process that might be used to intervene in the process. Furthermore, this integrated analysis implicates pre-existing drugs that might be re-purposed to help prevent or reduce metastatic burden in patients. The combined results obtained from this analytical strategy therefore provide an important platform for further genome-wide analysis into the etiology of metastatic disease.

## Introduction

Metastasis is an extremely complex process that involves not only tumor-autonomous events but also interactions with local microenvironment and distant tissues. Hundreds or thousands of genes are thought to be associated with metastatic progression [1]; however, the proportion of genes that contribute etiologically to tumor progression is unknown. Identification of genes that contribute mechanistically to metastasis will deepen our understanding of tumor progression and potentially provide novel targets for prevention or improving patient outcome. Because metastatic disease is the major cause of patient mortality and morbidity for patients with solid tumors [2], the ability to prevent or successfully intervene would be expected to demonstrate significant clinical benefit. Hence strategies to accelerate metastasis-associated gene discovery are particularly valuable for understanding terminal stages of neoplastic disease.

**Fig 1:**
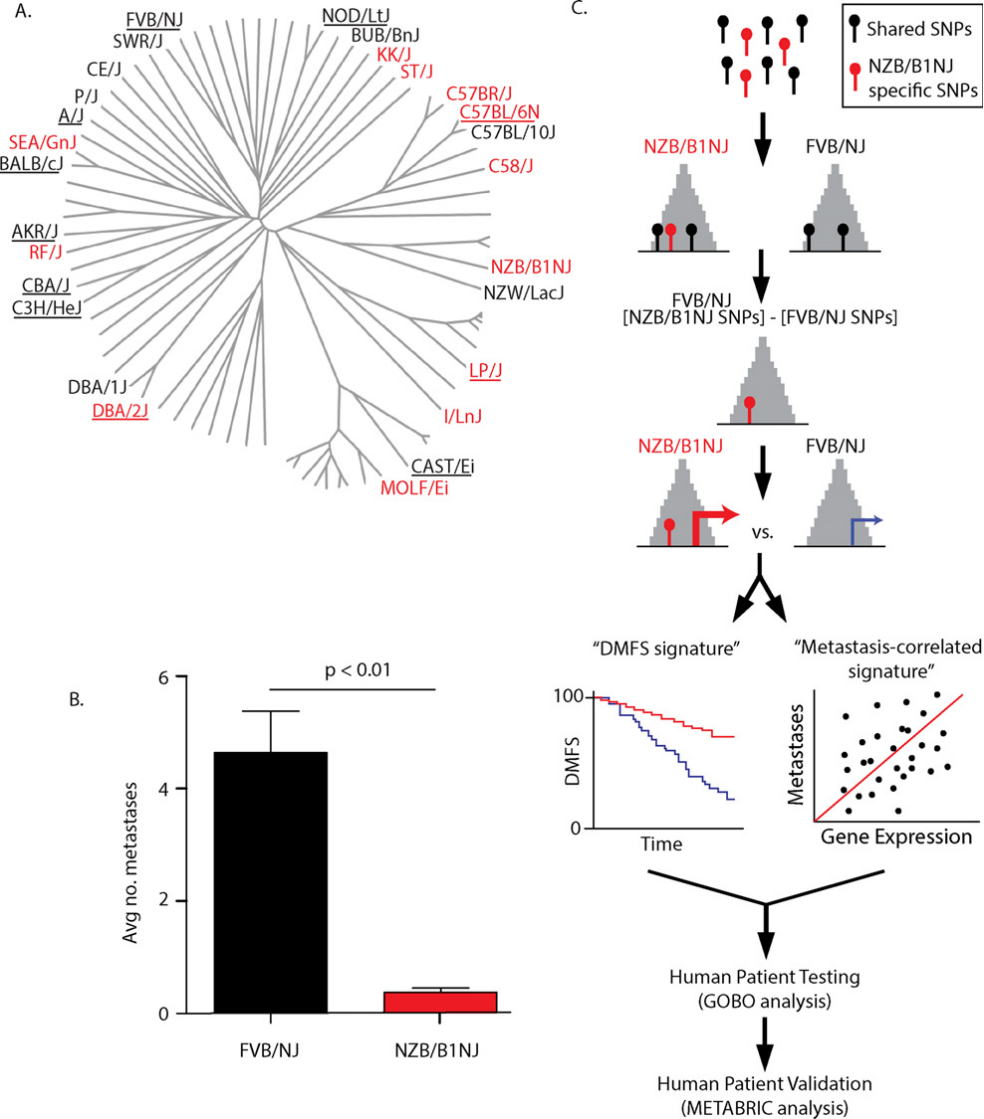
Identifying metastasis-associated genes using an integrated subtractive approach. A) Phylogenetic tree showing the inbred strains used in the original metastasis susceptibility screen and their phylogenetic relationship. Black labels indicate strains that were not significantly different from the original FVB/NJ MMTV-PyMT background. Red labels were strains that show significant reductions in pulmonary metastases. Underlined strains indicate strains sequenced by the Welcome Trust. The phylogenetic tree is adapted from Genetics (2010) v185 pgs 1081–1095 [6]. B) Comparison of the metastatic capacity the MMTV-PyMT FVB/NJ, and NZB/B1NJ genotypes. The p value for the comparison between FVB/NJ and NZB/B1NJ metastatic capacity was calculated by ANOVA analysis corrected for multiple testing across the entire set of strains depicted in panel A. C) Schematic representation of the low stringency subtractive strategy to enrich for genes associated with metastatic progression. Grey peaks represent DNAse hypersensitivity sites.

Our laboratory previously demonstrated that different inbred mice lineages (S1 Fig) possess different propensities for metastatic disease. The highly metastatic FVB/NJ-TgN(MMTV-PyMT)^634mul^ model [3] (MMTV-PyMT) was bred to 27 inbred strains and significant suppression of metastasis was observed in the progeny of 12 inbred strains. Since all of the tumors were induced by the same transgene, this suggests that polymorphisms in the genetic background can influence metastatic progression [4] (S1 Fig). Interestingly, projecting the metastatic capacity of each strain onto the mouse phylogenetic tree demonstrated closely related strains can have distinct metastatic capacities (Fig 1A). This observation suggests that single nucleotide polymorphisms (SNPs) which distinguish these closely related inbred strains are enriched for factors causally associated with metastatic progression. As a consequence, genome-wide identification of these SNPs and their associated genes provide a rapid method for capturing significantly more metastasis-associated genes than previous single candidate gene genetic strategies (e.g. Ref. [5]). Herein we describe a novel integrative strategy to identify candidate metastasis-associated genes based on an integrated mouse-human genome-wide inherited susceptibility systems genetics screen.

## Results

### An integrated subtractive approach to identify metastasis-associated genes

To test this strategy, whole genome sequencing of NZB/B1NJ was performed. This strain was selected because of the availability of pre-existing linkage, expression, and validated metastasis susceptibility gene data, enabling internal validation of the screen [4, 5, 7, 8]. Comparison of metastatic capacity demonstrated a significant suppression of metastasis by the NZB/B1NJ genome compared to the original FVB/NJ background of the MMTV-PyMT animal (Fig 1B). (Raw data: ERP000927; polymorphism data available at http://www.sanger.ac.uk/resources/mouse/genomes/). After alignment of the reads to the C57BL/6J mouse reference genome (GRCm38), approximately 5 million SNPs that distinguish NZB/B1NJ were identified, including approximately 54,000 unique SNPs.

A number of observations suggest that a subtractive strategy integrated with epigenetic and transcriptional filters could identify SNPs potentially driving metastatic disease. First, analysis of SNPs associated with phenotypes in human GWAS demonstrated that 88% of these SNPs are intronic or intergenic [9] and 71% fall within DNAse hypersensitivity sites (DHS) [10] which delineate *cis*-regulatory elements (promoters, silencers, insulators, enhancers, locus control regions) [11]. Together these data suggest that more than half of GWAS-associated SNPs are associated with polymorphic DHS (pDHS). Consequently, DHS represent less than 1% of the mouse genome, and only 10% of pDHS are associated with nearby transcriptional variation [12].

As most inherited variation is thought to result from changes in gene expression rather than structural changes in proteins, we restricted our analysis to those SNPs within the DHS sites [13] in the well-defined mouse mammary adenocarcinoma 3134 cell line [14, 15]. This reduced the number of SNPs for consideration from more than 5 million to approximately 120,000. Further, the SNPs shared between NZB/B1NJ and FVB/NJ [16] (the host genome for the MMTV-PyMT transgene) were subtracted from the overall list to enrich for SNPs likely to be causally associated with the reduced metastatic susceptibility observed in the NZB/B1NJ strain. The total number of NZB/B1NJ SNPs that remained after the subtraction was 54,659 (Fig 1C, S1 Table). The SNPs were then mapped to genes using the Genomic Regions Enrichment of Annotations Tool (GREAT) [17] using the default association rules. A total of 7902 genes were associated with pDHS following this analysis.

Next, the genes were screened across two panels of mouse mammary tumors to enrich for those genes with pDHS that alter gene expression. These panels were generated from crossing the MMTV-PyMT transgenic mammary tumor model and the highly genetically diverse mouse genetic mapping panel, the Diversity Outbred (DO) population [18]. This population is a randomly bred population generated from 8 progenitor inbred strains, including wild-derived representatives of the major mouse subspecies, *Mus musculus*, *Mus domesticus*, and *Mus castaneous*. As a consequence, the DO population closely resembles natural populations like humans [19] and is therefore likely to capture much of the heritable expression level variation within the *Mus* genus.

The two populations of tumors were generated from crosses between the MMTV-PyMT and different subsets of animals from the 5^th^ generation (G5 N = 131) [20] or 7^th^ generation (G7 N = 159) of the DO population. Approximately 25% of the total DO population was used to breed with MMTV-PyMT for each generation (45 females out of 175 DO breeding cages). As a result, these two populations of DO mice were expected to carry distinct, yet overlapping combinations of SNPs. Consistent with this, the tumor phenotypes were significantly different between the two mouse populations (ex. G5: 74/129 mice with metastatic disease; G7: 132/161 mice with metastatic disease; p = 4.4x10^-8^; S2 Fig). We therefore chose to screen the populations separately for metastasis-associated genes.

To investigate the role of polymorphism on transcription, total RNA from the mammary tumors was assayed on Affymetrix ST v1.0 chips. The 7902 genes with polymorphic DHS were tested for significant expression variation across each of the DO populations. Genes that exhibited significant variation (p<0.05) within each DO x PyMT population were assumed to have polymorphisms that functionally affected transcription and were included for further analysis. Genes without significant expression variation across the DO populations were assumed to have SNPs that did not affect gene transcription and were excluded from further analysis. This filter reduced the metastasis-associated candidate gene list to 2810 genes for the DO G5 tumors and 3223 genes for the DO G7 tumors. These differentially expressed genes were then subjected to analysis using BRB ArrayTools survival or quantitative trait tools to identify genes from each of the DO data sets associated with metastatic disease. The resulting screen yielded 4 lists of potential metastasis susceptibility genes ranging from 358–1518 members (See Table 1 and S2 Table). Examination of the signatures indicated that only a minority of the genes were common between the *DMFS* (*distant metastasis-free survival*) and *metastasis-correlated signatures* (S3 Fig), consistent with the two DO populations comprising different combinations of metastasis-associated factors.

**Table 1:** Number of genes identified by screening methods.

		Number of genes	Prognostic for DMFS in GOBO	Prognostic for OS in GOBO
DO Cross	Genes with pDHS	7902	ND	ND
G5	Number of variably expressed genes	2810	ND	ND
	DMFS genes	441	Y	N
	Metastasis correlated genes	358	Y	Y
G7	Number of variably expressed genes	3223	ND	ND
	DMFS genes	1518	Y	Y
	Metastasis correlated genes	764	Y	Y

### Metastasis-associated candidate genes predict outcome in human breast cancer

**Fig 2:**
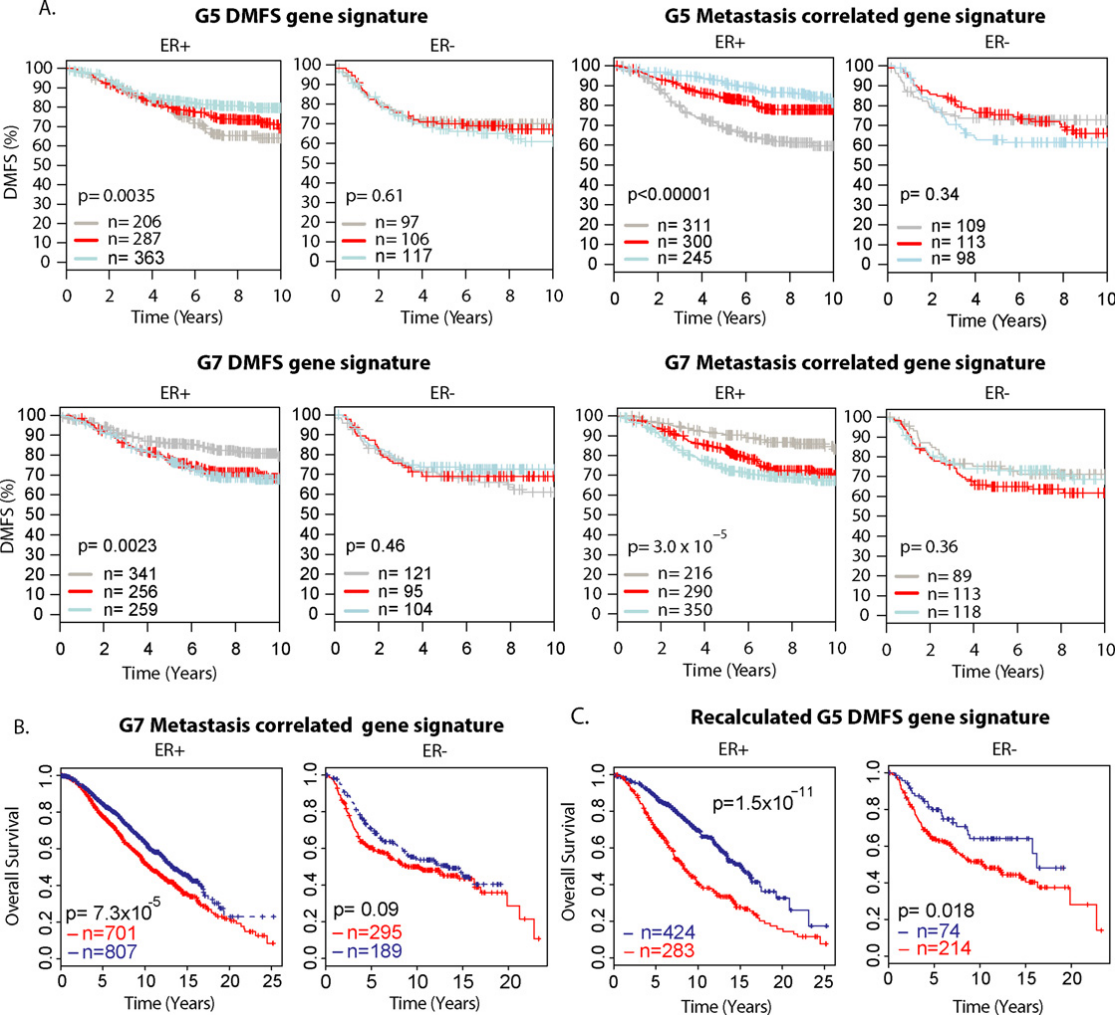
Metastasis-associated candidate genes predict outcome in human breast cancer. A) Distant metastasis-free survival analysis of the DMFS gene signatures (left panels) or the metastasis correlated gene signatures (right panels) on the GOBO data sets. Estrogen receptor status for each subset of patients is indicated above the Kaplan-Meier plots. B) Overall survival analysis of the mouse G7 metastasis-correlated gene signatures on the METABRIC patient data set. C) Performance of the G5 DMFS signature on the METABRIC validation data set after recalculation of gene weights on the METABRIC discovery data set.

We next evaluated the performance of this strategy on a genome-wide basis. The prognostic ability of each of the gene signatures was tested on human breast cancer datasets. The signature hazard ratio (DMFS signature) or the correlation coefficient (metastasis-correlated signature) from the mouse data provided weight and direction to each gene to require identical functionality between the two species. The weighted gene signatures were then screened for their ability to discriminate outcome in human breast cancer using the Gene expression-based Outcome for Breast cancer Online (GOBO) tool [21]. Of interest, all four signatures were prognostic in estrogen receptor-positive (ER^+^) but not estrogen receptor-negative (ER-) tumors (Fig 2A) in the GOBO data set, suggesting that a significant fraction of genes in the signatures contribute in the same manner to metastatic progression in both species. For a second independent validation, the signatures were then tested on the METABRIC gene expression dataset [22]. The weighted metastasis-correlated signatures were also prognostic in the ER+ subset in this patient dataset (Fig 2B and S4 Fig). The DMFS signatures did not discriminate outcome in this dataset.

Of note, outcome in the GOBO dataset is distant metastasis-free survival (DMFS) and a large fraction of the data is from adjuvant treatment-naïve patients, similar to the treatment-naïve mouse populations. In contrast, the METABRIC outcome data is overall survival and includes response to neo-adjuvant, adjuvant, and salvage therapy, all of which can alter the clinical course of disease for a significant fraction of patients [23], and may subsequently change the relative weights of genes within the signature. The weight for the G5 DMFS signature gene was therefore recalculated using half of the METABRIC data (discovery set) and validated on the remaining samples (test set) (Fig 2C). This analysis indicates that the genes identified by the DMFS screen are associated with tumor progression in mouse and humans, although the relative weight of each contributing gene may not be preserved. Results showed both gene signatures can better discriminate outcome in ER+ versus ER- breast tumors, which is consistent with the MMTV-PyMT being a model for human luminal breast cancers [24, 25].

To confirm that these gene signatures were more prognostic then thos generated by chance, three different analyses were performed using the METABRIC data set. In the first set of analyses, the weights were held constant and random genes were assigned for the 656 genes of the metastasis-correlated signature and the 383 genes of the recalculated METABRIC DMFS signature. The process was permuted 1000 times and the performance of the randomly assigned genes was compared to the experimentally derived signatures. Both of the experimentally performed signatures were significantly better at discriminating outcome in the METABRIC data than the weighted signatures for randomly assigned genes (for ER+ tumors p = 0.035 for the metastasis-correlated signature, p <0.001 for the DMFS signature).

Next, the identity of genes within the metastasis-correlated and DMFS signatures were held constant, but the weights were randomly generated by the pseudo random generator following normal distribution. This process was also permuted 1000 times and compared to the performance of experimentally derived signatures. The experimentally derived signatures also outperformed the signatures with random weights (for ER+ tumors p = 0.048 for the metastasis- correlated signature, p < 0.001 for the DMFS signature). Finally, we generated random weights with normal distribution and randomly selected gene sets of size 656 and 383 respectively and tested the random signatures 1000 times. Once again, the experimentally derived signatures were significantly better than the permuted data (for ER+ tumors p = 0.028 for the metastasis- correlated signature, p < 0.001 for the DMFS signature). Of note, the experimentally recalculated *DMFS* signature was also significantly better than the permuted signatures under all three conditions for patients with ER- as well as ER+ tumors (S3 Table). These data suggest that the genes identified by the integrated mouse subtractive strategy were unlikely to have been implicated with metastatic disease by chance.

### Validation of the pDHS screen for metastasis QTL candidate genes

**Fig 3:**
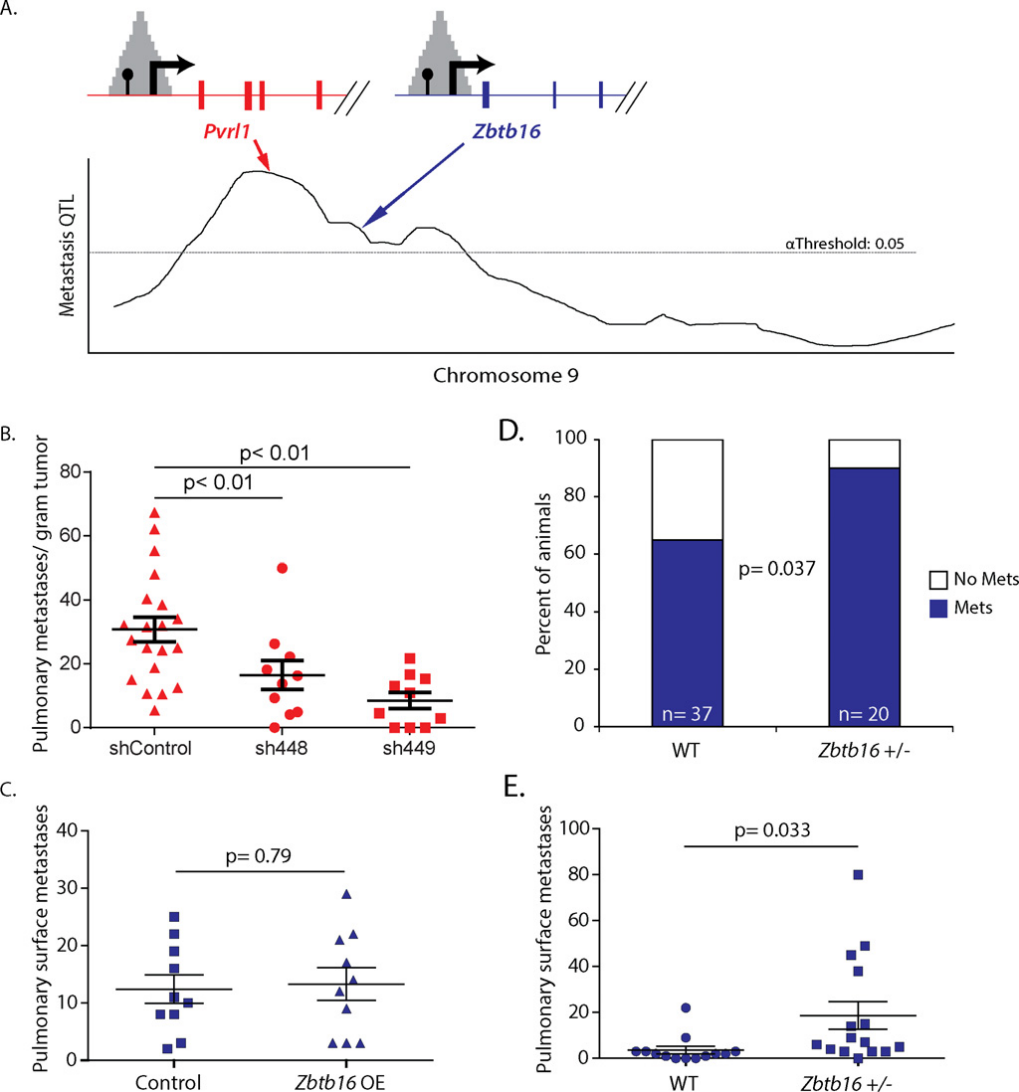
Functional analysis of *Pvrl1* and *Zbtb16* validates the pDHS method. A) Gene lists were overlaid on the Chromosome 9 metastasis modifier locus to identify *Pvrl1* and *Zbtb16*. B) Results of the spontaneous pulmonary surface metastasis assays after orthotopic implantation of Mvt1 (left panel) mammary tumor cells with *Pvrl1* knocked down. P values represent the result of an ANOVA test after Dunnetts correction for multiple comparisons against the shControl data. C) Results of the spontaneous pulmonary surface metastasis assays after orthotopic implantation of Mvt1 cells over-expressing *Zbtb16*. The p value was calculated using a two-sided parametric t test. D) Metastasis incidence analysis of MMTV-PyMT animals either homozygous wild type (WT) or heterozygous knockout (+/-) for *Zbtb16*. The p value was calculated by a two-sided Chi-square test. E) Results of the spontaneous pulmonary surface metastasis assays after orthotopic implantation of wildtype Mvt1 cells into animals either homozygous wildtype (WT) or heterozygous knockout (+/-) for *Zbtb16*. The p value was calculated using a two-sided parametric t test. Scatterplots are shown with mean values with standard error of mean. All statistical calculations were performed using GraphPad Prism v. 6.01.

To evaluate the subtraction strategy, the gene lists were compared with existing data. Linkage analysis previously identified the presence of a metastasis modifier locus on NZB/B1NJ chromosome 9 [7, 8], located 16 to 67 megabases distal to the centromere [5] (Fig 3A), containing approximately 1300 genes: ~ 800 annotated genes and ~ 500 predicted genes. Limiting the subtracted gene lists to this interval further reduced the number of candidates to 6 to 25 genes (Table 2). Encouragingly, one of the DMFS genes was *Cadm1*, a previously identified metastasis susceptibility gene [5]. For further validation, the gene closest to the modifier peak, *Pvrl1*, was tested. shRNA knockdowns of *Pvrl1* in two independent mouse mammary tumor cell lines was performed. Knockdown of *Pvrl1* had inconsistent effects on tumor growth and *in vitro* cell proliferation, but consistently reduced metastatic disease (S5 Fig). Normalization of metastatic burden by tumor weight to account for differences in tumor weight *in vivo* still resulted in significant differences between control and knockdown cells (Fig 3B), consistent with *Pvrl1* being a tumor progression gene.

**Table 2:** Number of Chr 9 genes identified by screening methods.

		Number of genes
DO Cross	Genes with pDHS	7902
G5	Number of variably expressed genes	2810
	Chr 9 DMFS genes	12
	Chr 9 Metastasis correlated genes	9
G7	Number of variably expressed genes	3223
	Chr 9 DMFS genes	6
	Chr 9 Metastasis correlated genes	25

To evaluate a candidate gene identified only in the G7 population screen, the gene *Zbtb16* was selected (Fig 3A). *Zbtb16* was previously implicated as a candidate due to its membership in a proliferation-associated gene network that is predictive of metastatic disease [26]. However, orthotopic implantation of cells with *Zbtb16* overexpression did not show any differences in metastatic disease (Fig 3C) suggesting *Zbtb16* might have a tumor cell non-autonomous effect. *Zbtb16* knockout animals [27] were therefore bred to MMTV-PyMT animals to generate PyMT^+^/*Zbtb16*^+/-^ or PyMT^+^/*Zbtb16*^+/+^ animals. *Zbtb16* heterozygotes showed increased incidence of metastasis (p = 0.04; Fig 3D), but did not display a statistically significant change in metastatic burden (S6A Fig), likely due to high experimental variability. Tumor weights from *Zbtb16+/-* animals were not significantly different compared to tumors from their wild type littermates (p = 0.82; S6B Fig) indicating the effect of *Zbtb16* was unlikely due to differences in tumor cell proliferation. Consistent with a tumor cell non-autonomous role, injection of wildtype mammary tumor cells into wildtype or *Zbtb16*^+/-^ animals showed a significant increase in pulmonary colonization in the heterozygous animals (Fig 3E) without significant effect on primary tumor burden (p = 0.81; S6C Fig).

To further validate this method, the genome-wide gene lists were examined to try and identifying metastasis-associated genes outside of the metastasis-associated susceptibility peaks. Analysis discovered a number of genes previously identified as playing important roles in metastatic disease (e.g., *Tpx2* [28], *Angptl4* [29], *Ezr* [30], *Txnip* [31]), indicating this strategy is capable of identifying putative metastasis genes on a genome-wide scale.

### Metastasis candidates implicate molecular functions in metastatic etiology

To identify the cellular and molecular pathways contributing to metastatic disease, Ingenuity Pathway Analysis (IPA) was performed. The most significant canonical pathways for genes associated with metastasis in the G5 DO population was antigen presentation for the *DMFS signature* (p = 4.7x10^-8^) and mitotic pathways for the metastasis-correlated signature (p = 1.28x10^-4^–1.07x10^-6^; S3 Table). These results are consistent with our [5] and other laboratories’ [32–34] findings showing a significant role for immunity or cellular proliferation in breast cancer progression. In contrast, significant pathways associated with metastatic progression in the G7 DO population included IL-17 pathways for the DMFS gene signature and metabolic and rheumatoid arthritis pathways for the metastasis-correlated genes, including diabetes signaling (p = 7.23x10^-4^–2.3x10^-4^; S4 Table).

IPA analysis was also performed to identify potential upstream regulatory genes that contribute to prognostic signatures and tumor progression. All four gene sets implicated TGFβ1 as an important upstream regulator that suppresses metastatic disease (p = 3.7x10^-3^–9.3x10^-18^), consistent with the role of TGFβ in early tumor progression [35]. Estrogen receptor-α and -β, and the progesterone receptor were also identified as significant upstream regulators in both analyses (S5 Table). These results further support the utility of the pDHS strategy in identifying metastasis-relevant pathways and implicate additional pathways for investigation.

### The pDHS screen implicates novel drugs for anti-metastatic therapy

Ingenuity Pathway Analysis of the gene lists was then carried out to identify potential clinically actionable targets or drugs for metastatic therapy. Tamoxifen, a current standard of care therapeutic for breast cancer treatment, was associated with suppression of metastatic disease in both the DMFS and metastasis-correlated analyses (S1 Table), suggesting this strategy can identify clinically relevant therapeutics. In addition, the cannabinoid receptor 1 gene, *CNR1*, was found to be associated with metastatic suppression (S5 Table). Consistent with this possibility, an independent study showed that synthetic cannabinoids suppress tumor growth and metastasis in the MMTV-PyMT model [36].

Moreover, the data suggest that 8-bromo-cAMP, a cell permeable cAMP analog, is associated with metastatic suppression (S5 Table). Increased cAMP levels are a downstream consequence of caffeine (a non-specific phosphodiesterase inhibitor [37]) metabolism and interestingly cAMP was previously shown in our laboratory to be a metastasis-suppressing agent [38]. Treatment of a highly metastatic mouse mammary tumor cell line with 8-bromo-cAMP induced a gene signature that was an independent predictor of both DMFS and overall survival in GOBO datasets (S7 Fig), indicating this pathway may be a useful clinical target.

**Fig 4:**
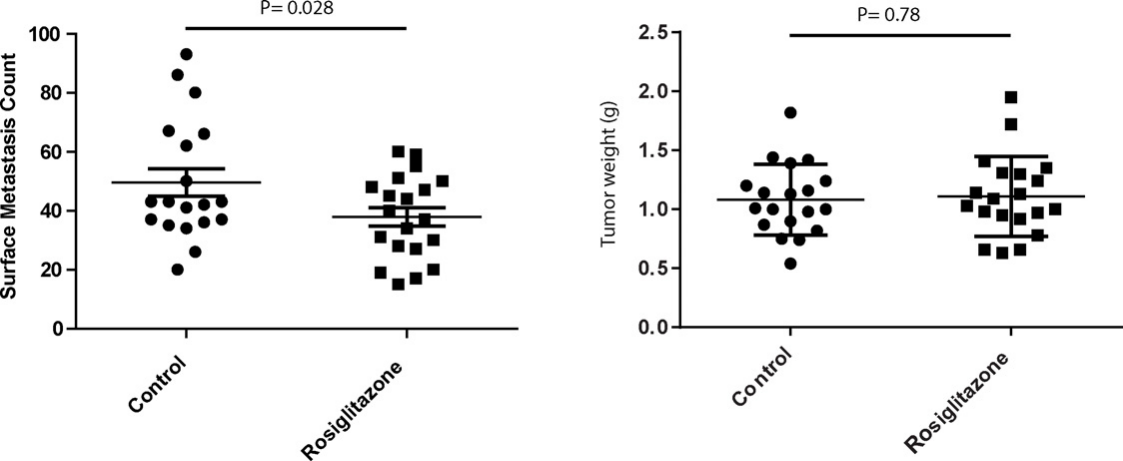
Effect of Rosiglitazone on pulmonary metastasis. Surface metastasis counts for animals implanted with the highly metastatic mouse mammary tumor cell line 6DT1, treated with either vehicle (control) or Rosiglitazone. P values were calculated using a two-sided parametric t test. Scatterplots are shown with mean values with standard error of mean. All statistical calculations were performed using GraphPad Prism v. 6.01.

Finally, the IPA analysis not only implicated the diabetes signaling pathway as significantly associated with metastatic disease, but also a number of agents used to treat diabetes that might suppress metastatic disease. To test this prospect within our experimental systems, metastatic mouse mammary tumor cells were implanted into immunocompetent mice. The mammary tumors were permitted to grow until established (10 days post-injection) and then the mice were treated with rosiglitazone until euthanasia (28 days post-injection). As predicted from the integrated analysis, the rosiglitazone-treated group had 25% fewer pulmonary metastases compared to the control group (Fig 4; p = 0.028), consistent with previous reports for the LMM3 mammary tumor cell line [39].

## Discussion

We have integrated quantitative trait genetics, transcriptional epigenetics, gene expression, and computational biology tools in a mouse model of metastatic mammary cancer to identify factors that contribute to inherited susceptibility to breast cancer metastasis. This approach significantly enriched for genes associated with metastatic disease as measured by the generation of gene signatures with prognostic value in human patients. Interestingly, our analyses has resulted in signatures that are prognostic in both early relapse and late relapse clinical scenarios (Fig 2A). The majority of gene expression signatures currently available for human breast cancer patients are limited because they are only associated with early relapse, hence this type of integrated mouse-human analysis could complement existing data as it provides additional insights into the later stages of metastatic disease.

Importantly, all of the gene signatures described herein are weighted and must contain genes that transcribe in the same direction with equal relative potency in both mouse and human. The ability of the mouse signatures to maintain prognostic ability in human samples supports the assumption that the underlying biology of metastasis is very similar between these two species. If this were not the case, one would not expect a weighted signature to preserve its prognostic ability across species. These results therefore provide additional support for the application of metastatic mouse models to characterize and identify metastasis genes.

Our results also illustrate an important caveat to this hypothesis, described as such: because genetic and molecular analysis was performed using a treatment-naïve mouse model, the results should represent the natural course of the disease. In contrast, patients are commonly treated using surgical resection of the primary tumor combined with subsequent radiation and adjuvant therapies to reduce the risk of recurrence. Since adjuvant therapy reduces the number of patients who develop metastatic disease by 20–30% [40] and salvage therapy prolongs survival for patients who develop metastasis, the inability of the DMFS signature to predict overall survival in treated METABRIC patients without re-optimization is not surprising. Furthermore, the gene signature difference between treatment-naïve and treated patients may also contribute to the conflicting association results for SIPA1, which is associated with treatment-naïve DMFS samples [41, 42], but not overall survival [43]. Hence accounting for the two different phenotypes (treatment-naïve DMFS vs. overall survival) is an important consideration when translating mouse model data into clinical useful human observations.

Furthermore, the gene signatures and associated gene lists derived from this analysis should be enriched for metastasis-associated genes. Prognostic gene signatures are usually derived by correlating gene expression with metastatic disease without distinguishing whether the associated gene expression is etiological or a secondary effect. The requirement for candidate genes to have polymorphisms within putative enhancers and promoters should significantly enrich for genes with primary effects. The ability of this strategy to both re-identify known metastasis susceptibility genes (e.g., *Cadm1*) and validate novel tumor progression genes (e.g., *Pvrl1*, *Zbtb16*) is consistent with this hypothesis. The resulting gene lists will therefore provide greater utility for identifying molecular and cellular pathways associated with the metastatic process.

Finally, the integrated strategy described herein allows for the identification of novel agents with anti-metastatic therapy potential. In addition to re-identifying agents that are the current standard of care (tamoxifen) and validating experimental drugs (cannabinoids [36]), this technique also identified FDA approved anti-diabetic drugs as potentially useful therapeutics. Of interest, orthotopic implantation assays confirmed the ability of one of these drugs, rosiglitazone, to reduce metastatic burden [39]. Investigations of other agents used to treat type II diabetes is also supported by the results obtained in this metastatic mammary tumor model. Increased survival in ER+/HER2+ breast cancer patients [44, 45] and triple-negative breast cancer [46] treated with the anti-diabetic drug metformin have been reported, as well as investigations regarding its ability to increase survival in other forms of malignancy [47]. These data support the ability of this integrated analysis to identify potentially useful tools for clinical use and further support the complementary use of animal models systems to understand the complex biology of metastatic disease.

## Materials and Methods

### Ethics statement

The research described in this study was performed under the Animal Study Protocols LPG-002 and LCBG-004, approved by the NCI Bethesda Animal Use and Care Committee. Animal euthanasia was performed by cervical dislocation after anesthesia by Avertin.

### Whole genome sequencing

NZB/B1NJ and NZW/LacJ DNA was obtained from The Jackson Laboratory DNA Repository. Library preparation and sequencing on Illumina HiSeq instruments was performed following the manufacturers recommended protocols. Four lanes of 102 base paired end sequence per strain was performed to achieve approximately 40x coverage. Reads were aligned to the reference genome (GRCm38) using BWA version 0.7.5a-r406 [19451168]. All lanes from the same library were then merged into a single BAM file using Picard tools and PCR duplicates were marked using Picard 'MarkDuplicates' [19505943]. Local realignment was carried out using GATK-v3.0 using default parameters to generate the set of intervals for realignment [20644199]. SNP and indel discovery was performed with the Samtools v1.1 (samtools mpileup -t DP,DV,DP4,SP,DPR,INFO/DPR -E -Q 0 -pm3 -F0.25) and calling was performed with Bcftools call v1.1 (bcftools call -mv -f GQ,GP -p 0.99). Raw sequencing data is available under accession ERP000927.

### DNAse hypersensitivity site and whole genome sequence datasets

DNase hypersensitive site (DHS) data for the mouse 3134 mammary adenocarcinoma cells was downloaded from the Encode project websites.

http://hgdownload.cse.ucsc.edu/goldenPath/mm9/encodeDCC/wgEncodeUwDnase/

http://hgdownload.cse.ucsc.edu/goldenPath/mm9/encodeDCC/wgEncodeUwDgf/

Genotype data for FVB/NJ was downloaded from the Welcome Trust Sanger Centre website: http://www.sanger.ac.uk/resources/mouse/genomes/ The NZB/B1NJ and NZW/LacJ genotype data is also available at this site.

### Computing environment

All computations were performed on NIH helix/biowulf system, documentation of which is available at https://helix.nih.gov. We used R computing environment, perl scripts, bedtools, and ucsc liftOver for most of the analyses.

### Identification of polymorphic DHS sites

The workflow consisted of the following. The UCSC liftOver tool was used to convert between mm9 and mm10 as necessary when using bedtools intersectBed to intersect two bed files. 1) The Encode DHS data were filtered for the regions overlapping with polymorphic sites. Since the DHS data were generated in Genome Build mm9, we used UCSC mm9 snp128 data to restrict the DHS sites. 2) The mice NZB/B1NJ genotype data in vcf was filtered to retain the SNPs that overlap with the DHS present in the 3134 cells. 3) We then removed SNPs in the DHS that are present in the mouse FVB/NJ strain.

### Genomic Regions Enrichment of Annotations Tool (GREAT) analysis

BED files containing the subtracted NZB/B1NJ specific, DHS associated SNPs were loaded into the GREAT tool website using the default settings for gene assignment. GREAT calculates statistics by associating genomic regions with nearby genes and applying the gene annotations to the regions. Association is a two-step process. First, every gene is assigned a regulatory domain. Then, each genomic region is associated with all genes whose regulatory domain it overlaps. The default association settings included assignment of basal regulatory elements 5 kb upstream and 1 kb downstream of transcriptional start sites (regardless of other nearby genes). In addition, the gene regulatory domain was extended up to 1 megabase in both directions to the nearest gene's basal domain but no more than the maximum extension in one direction [16].

### DO ST array analysis

The Diversity Outcross x MMTV-PyMT G5 CEL files have been deposited in the Gene Expression Omnibus (GEO) database (www.ncbi.nlm.nih.gov/geo) under the accession number GSE48565. The G7 CEL files are deposited under the accession number GSE64522. Data was imported into BRB ArrayTools version 4.3.2 (http://brb.nci.nih.gov/BRB-ArrayTools/) and normalized using the median array as a reference. Batch correction was not performed before BRB ArrayTool analysis. Only genes with a Log Intensity Variation of p< 0.05 were considered for further analysis. Distant metastasis free survival analysis of these data was performed using the Find Genes Associated with Survival tool (DMFS genes). Genes were considered associated with DMFS if p< 0.05. Genes correlated with metastatic disease was performed using the Spearman Correlation Test option of the Find Genes Associated with Quantitative Trait tool (metastasis correlated genes). Genes with a p< 0.05 were considered to be significantly associated with metastatic disease.

#### Gene signature analysis using the GOBO webtool

To provide direction and relative importance to each gene, weights for each gene in the signature was determined from the DO mouse crosses. For the DMFS signature, the hazard ratios associated with DMFS in the DO G5 cross was log2 transformed to provide direction and relative strength for each gene in the signature. For the metastasis-correlated signature, the correlation coefficient for each gene with metastasis number in the DO G7 data was used. The weighted gene was then uploaded to the GOBO tool and analyzed using the default settings for the ER+ and ER- subsets of breast cancers. P values reported are those generated by the GOBO analytical tool.

### 8-Br-cAMP microarray analysis

2x10^5^ Mvt1[48] mammary tumor cells were plated in 6 well dishes. 24 hours later the cells were treated with 500 uM 8-Br-cAMP (Sigma) dissolved in 0.1% DMSO for 24 hours, then RNA harvested using Trizol following the manufacturers recommended protocol. Vehicle control or 8-Br-cAMP samples were performed in triplicate. Transcriptome analysis performed by the NCI Laboratory of Molecular Technology using Affymetrix MOE430 v2 chips. The data was analyzed using the Class Comparison tool of BRB ArrayTools. For the gene signature analysis, the differentially expressed genes were filtered for those with greater than 10-fold change in expression, either up- or down-regulated, compared to the vehicle alone. The fold change in gene expression was used to weight the individual genes, and the ability of the weighted gene signature to discriminate breast cancer patient outcome assessed using the webtool GOBO (http://co.bmc.lu.se/gobo/).

### Cell lines

The 6DT1 and Mvt1 cell lines[48] were obtained from the laboratory of Robert Dickson, George Washington University. Microsatellite genotyping validated that these cell lines originated from an FVB/NJ animal. Both cell lines have been demonstrated to be mycoplasma-free.

### Pvrl1 knockdown experiment

shRNA lentiviral vectors were purchased from Sigma-Aldrich (cat. # SHCLNG-NM_021424). Stable *Pvrl1* knockdown cell lines were generated by lentiviral transduction into Mvt1 cells and knockdown validated by qRT-PCR. 8x10^5^ Mvt1 or 10^5^ 6DT1 cells were inoculated into the four mammary fat pad of 6–8 week old FVB/NJ female mice, 10 animals per group. Animals were euthanized at 5 weeks (experiment 1) or 4 weeks (experiment 2) after implantation. One-way Anova with Dunnett’s correction for multiple testing was performed for each experiment using GraphPad Prism. The results of the replicate experiments were then combined using Fisher’s combined probability test. All procedures were performed as approved by the NCI-Bethesda Animal Care and Use Committee.

### Zbtb16 validation experiment

Epitope-tagged *Zbtb16* over-expression was performed by lentiviral transduction into the cell line Mvt1. Orthotopic implantation was carried out as described above and analyzed using the Mann-Whitney test in GraphPad Prism. Autochthonous tumor assays were performed by breeding MMTV-PyMT male animals to *Zbtb16*^+/-^ female animals to generate PyMT^+^/*Zbtb16*^*+/-*^ female animals. 20 PyMT^+^/*Zbtb16*^*+/-*^ animals was selected for analysis based on previous experience which suggested this as an appropriate group size to achieve statistical significance. Statistical differences were assessed using the Mann-Whitney test in GraphPad Prism. All procedures were performed as approved by the NCI-Bethesda Animal Care and Use Committee.

### Effect of Rosiglitazone treatment on pulmonary metastases

100,000 cells/mouse of the mammary tumor cell line 6DT1 were orthotopically implanted into the fourth mammary fat pad of female FVB/NJ mice (6–8 weeks old). 10 days post-implantation all of the animals were combined into a single cage then randomly assigned to treatment or control group by alternating assignment to new cages. Roziglitazone (100μM) or vehicle (DMSO, 0.17%) was added to drinking water 10 days post tumor implantation and available to mice ad libitum. Drinking water with Roziglitazone or DMSO was refreshed every week. Tumor growth was monitored and animals were euthanized 28 days post implantation. Tumors and lungs were evaluated for weight and surface metastases respectively. The experiment was performed twice and the results of the Mann-Whitney test combined using Fisher combined probability test. All procedures were performed as approved by the NCI-Bethesda Animal Care and Use Committee.

### Data access

NZB/B1NJ sequence polymorphism data available data is available at http://www.sanger.ac.uk/resources/mouse/genomes/ Raw data is available at http://sra.dnanexus.com/studies/ERP000927. The Diversity Outcross x MMTV-PyMT G5 CEL files have been deposited in the Gene Expression Omnibus (GEO) database (www.ncbi.nlm.nih.gov/geo) under the accession number GSE48565. The G7 CEL files are deposited under the accession number GSE64522.

## Supporting Information

S1 Fig Metastatic efficiency of the strains from the metastasis susceptibility screen. The original FVB/NJ MMTV-PyMT genetic background is indicated by the gold histogram bar. Strains not significantly different from MMTV-PyMT are indicated in blue. Stains that significantly suppress metastasis are indicated with red bars. The number of animals for each genotype is indicated under the X-axis.
(TIF)

S2 Fig Analysis of the tumor phenotypes for the Diversity Outcross x PyMT G5 and G7 population F1 animals. (TIF)

S3 Fig Venn Analysis of the overlap between the DMFS and metastasis-correlated signatures for the low and high stringency screens. (TIF)

S4 Fig Results of the Low Stringency weighted gene signatures analysis on the METABRIC gene expression data. Each gene signature was tested separately on the estrogen receptor-positive (ER+) or estrogen receptor-negative (ER-) subsets of patient data sets.
(TIF)

S5 Fig Results of the spontaneous pulmonary surface metastasis assays after orthotopic implantation of 6DT1 mammary tumor cells with knocked down of *Pvrl1*. A) *In vitro* qRT-PCR analysis of shRNA knockdowns of *Pvrl1* in Mvt1 and 6DT1 cells showing the relative expression in the knockdown cells compared to shScramble controls. B) *In vivo* qRT-PCR analysis of shRNA knockdowns of *Pvrl1* in Mvt1 and 6DT1 implanted tumors. N = 5 for each group. C) *In vitro* proliferation assays for the shRNA knockdowns in Mvt1 and 6DT1 cells as measured on the Incucyte ZOOM instrument. D) Pulmonary surface metastases and orthotopic tumor weight results for mammary fat pad implantation of *Pvrl1* shRNA knockdown Mvt1 and 6DT1 cells. P values represent the result of an ANOVA test after Dunnetts correction for multiple comparisons against the shControl data.
(TIF)

S6 Fig Comparison of the number of pulmonary metastases observed in MMTV-PyMT animals either homozygous wildtype (WT) or heterozygous knockout (+/-) for *Zbtb16*. A) Pulmonary surface metastasis in PyMT animals either wild type (WT) or heterozygous (Zbtb16+/-) for a Zbtb16 knockout. B) Aggregate tumor weight in PyMT animals either wild type (WT) or heterozygous (Zbtb16+/-) for a Zbtb16 knockout. C) Tumor weights of wild type (control) or Zbtb16 over expressing (Zbtb16 OE) tumor cells orthotopically implanted into the fourth mammary fat pad of FVB/NJ mice. The p value was calculated using a two-sided parametric t test.
(TIF)

S7 Fig Distant metastasis-free survival (left panel) and overall survival (right panel) of the GOBO breast cancer patients based on the gene signatures induced by treatment of mouse mammary tumor cells with 8-Br-cAMP. (TIF)

S1 Table Number of SNPs present for consideration during subtraction process. (XLS)

S2 Table Gene symbols from low stringency (LS) and high stringency (HS) subtraction filtering. (XLS)

S3 Table Top canonical pathways identified by Ingenuity Pathway Analysis for genes associated with distant metastasis free survival (DMFS) or metastasis correlated gene analyses. (XLS)

S4 Table Upstream regulatory genes for the low and high stringency filtered gene signatures, as determined using Ingenuity Pathway Analysis tools. (XLS)

S5 Table Upstream drug analysis for the gene signatures, as determined using the Ingenuity Pathway Analysis tools. (XLS)

## References

[1] Ein-Dor L, Kela I, Getz G, Givol D, Domany E. Outcome signature genes in breast cancer: is there a unique set? Bioinformatics. 2005;21(2):171–8. 15308542 10.1093/bioinformatics/bth469

[2] Chaffer CL, Weinberg RA. A perspective on cancer cell metastasis. Science. 331(6024):1559–64. Epub 2011/03/26. 10.1126/science.1203543 21436443

[3] Guy CT, Cardiff R.D., and Muller W.J. Induction of mammary tumors by expression of polyomavirus middle T oncogene: A transgenic mouse model for metastatic disease. MCB. 1992;12:954–61. 1312220 10.1128/mcb.12.3.954-961.1992 PMC369527

[4] Lifsted T, Le Voyer T, Williams M, Muller W, Klein-Szanto A, Buetow KH, et al Identification of inbred mouse strains harboring genetic modifiers of mammary tumor age of onset and metastatic progression. Int J Cancer. 1998;77(4):640–4. Epub 1998/07/29. 9679770 10.1002/(sici)1097-0215(19980812)77:4<640::aid-ijc26>3.0.co;2-8

[5] Faraji F, Pang Y, Walker RC, Nieves Borges R, Yang L, Hunter KW. Cadm1 is a metastasis susceptibility gene that suppresses metastasis by modifying tumor interaction with the cell-mediated immunity. PLoS Genet. 2012;8(9):e1002926 Epub 2012/10/03. 10.1371/journal.pgen.1002926 23028344 PMC3447942

[6] Kirby A, Kang HM, Wade CM, Cotsapas C, Kostem E, Han B, et al Fine mapping in 94 inbred mouse strains using a high-density haplotype resource. Genetics. 2010;185(3):1081–95. Epub 2010/05/05. 10.1534/genetics.110.115014 20439770 PMC2907194

[7] Lancaster M, Rouse J, Hunter K. Modifiers for mammary tumor latency, progression and metastasis are present on mouse chromosomes 7, 9 and 17. Mamm Genome. 2005;16(2):120–6. 15859357 10.1007/s00335-004-2432-y

[8] Hunter KW, Broman KW, Voyer TL, Lukes L, Cozma D, Debies MT, et al Predisposition to efficient mammary tumor metastatic progression is linked to the breast cancer metastasis suppressor gene Brms1. Cancer Res. 2001;61(24):8866–72. 11751410

[9] Hindorff LA, Sethupathy P, Junkins HA, Ramos EM, Mehta JP, Collins FS, et al Potential etiologic and functional implications of genome-wide association loci for human diseases and traits. Proc Natl Acad Sci U S A. 2009;106(23):9362–7. Epub 2009/05/29. 10.1073/pnas.0903103106 19474294 PMC2687147

[10] Bernstein BE, Birney E, Dunham I, Green ED, Gunter C, Snyder M. An integrated encyclopedia of DNA elements in the human genome. Nature. 2012;489(7414):57–74. Epub 2012/09/08. 10.1038/nature11247 22955616 PMC3439153

[11] Thurman RE, Rynes E, Humbert R, Vierstra J, Maurano MT, Haugen E, et al The accessible chromatin landscape of the human genome. Nature. 2012;489(7414):75–82. Epub 2012/09/08. 10.1038/nature11232 22955617 PMC3721348

[12] Hosseini M, Goodstadt L, Hughes JR, Kowalczyk MS, de Gobbi M, Otto GW, et al Causes and consequences of chromatin variation between inbred mice. PLoS Genet. 2013;9(6):e1003570 Epub 2013/06/21. 10.1371/journal.pgen.1003570 23785304 PMC3681629

[13] John S, Sabo PJ, Thurman RE, Sung MH, Biddie SC, Johnson TA, et al Chromatin accessibility pre-determines glucocorticoid receptor binding patterns. Nat Genet. 2011;43(3):264–8. Epub 2011/01/25. 10.1038/ng.759 21258342 PMC6386452

[14] Lowy DR, Rands E, Scolnick EM. Helper-independent transformation by unintegrated Harvey sarcoma virus DNA. J Virol. 1978;26(2):291–8. Epub 1978/05/01. 26810 10.1128/jvi.26.2.291-298.1978 PMC354067

[15] Ostrowski MC, Richard-Foy H, Wolford RG, Berard DS, Hager GL. Glucocorticoid regulation of transcription at an amplified, episomal promoter. Mol Cell Biol. 1983;3(11):2045–57. Epub 1983/11/01. 6318079 10.1128/mcb.3.11.2045-2057.1983 PMC370071

[16] Wong K, Bumpstead S, Van Der Weyden L, Reinholdt LG, Wilming LG, Adams DJ, et al Sequencing and characterization of the FVB/NJ mouse genome. Genome Biol. 2012;13(8):R72 Epub 2012/08/25. 10.1186/gb-2012-13-8-r72 22916792 PMC3491372

[17] McLean CY, Bristor D, Hiller M, Clarke SL, Schaar BT, Lowe CB, et al GREAT improves functional interpretation of cis-regulatory regions. Nature biotechnology. 2010;28(5):495–501. Epub 2010/05/04. 10.1038/nbt.1630 20436461 PMC4840234

[18] Churchill GA, Gatti DM, Munger SC, Svenson KL. The diversity outbred mouse population. Mamm Genome. 2012;23(9–10):713–8. Epub 2012/08/16. 10.1007/s00335-012-9414-2 22892839 PMC3524832

[19] Roberts A, Pardo-Manuel de Villena F, Wang W, McMillan L, Threadgill DW. The polymorphism architecture of mouse genetic resources elucidated using genome-wide resequencing data: implications for QTL discovery and systems genetics. Mamm Genome. 2007;18(6–7):473–81. Epub 2007/08/04. 17674098 10.1007/s00335-007-9045-1 PMC1998888

[20] Hu Y, Bai L, Geiger T, Goldberger N, Walker RC, Green JE, et al Genetic background may contribute to PAM50 gene expression breast cancer subtype assignments. PLoS One. 2013;8(8):e72287 Epub 2013/09/10. 10.1371/journal.pone.0072287 24015230 PMC3756056

[21] Ringner M, Fredlund E, Hakkinen J, Borg A, Staaf J. GOBO: gene expression-based outcome for breast cancer online. PLoS One. 2011;6(3):e17911 Epub 2011/03/30. 10.1371/journal.pone.0017911 21445301 PMC3061871

[22] Curtis C, Shah SP, Chin SF, Turashvili G, Rueda OM, Dunning MJ, et al The genomic and transcriptomic architecture of 2,000 breast tumours reveals novel subgroups. Nature. 2012;486(7403):346–52. Epub 2012/04/24. 10.1038/nature10983 22522925 PMC3440846

[23] Singh A, Loscalzo J, Brigham and Women's Hospital. The Brigham intensive review of internal medicine New York: Oxford University Press; 2012. p. p.

[24] Herschkowitz JI, Simin K, Weigman VJ, Mikaelian I, Usary J, Hu Z, et al Identification of conserved gene expression features between murine mammary carcinoma models and human breast tumors. Genome Biol. 2007;8(5):R76 Epub 2007/05/12. 17493263 10.1186/gb-2007-8-5-r76 PMC1929138

[25] Pfefferle AD, Herschkowitz JI, Usary J, Harrell JC, Spike BT, Adams JR, et al Transcriptomic classification of genetically engineered mouse models of breast cancer identifies human subtype counterparts. Genome Biol. 2013;14(11):R125 Epub 2013/11/14. 10.1186/gb-2013-14-11-r125 24220145 PMC4053990

[26] Hu Y, Wu G, Rusch M, Lukes L, Buetow KH, Zhang J, et al Integrated cross-species transcriptional network analysis of metastatic susceptibility. Proc Natl Acad Sci U S A. 2012;109(8):3184–9. Epub 2012/02/07. 10.1073/pnas.1117872109 22308418 PMC3286991

[27] The International HapMap Project. Nature. 2003;426(6968):789–96. Epub 2003/12/20. 14685227 10.1038/nature02168

[28] Geiger TR, Ha NH, Faraji F, Michael HT, Rodriguez L, Walker RC, et al Functional analysis of prognostic gene expression network genes in metastatic breast cancer models. PLoS One. 2014;9(11):e111813 Epub 2014/11/05. 10.1371/journal.pone.0111813 25368990 PMC4219783

[29] Padua D, Zhang XH, Wang Q, Nadal C, Gerald WL, Gomis RR, et al TGFbeta primes breast tumors for lung metastasis seeding through angiopoietin-like 4. Cell. 2008;133(1):66–77. Epub 2008/04/09. 10.1016/j.cell.2008.01.046 18394990 PMC2390892

[30] Khanna C, Wan X, Bose S, Cassaday R, Olomu O, Mendoza A, et al The membrane-cytoskeleton linker ezrin is necessary for osteosarcoma metastasis. Nat Med. 2004;10(2):182–6. Epub 2004/01/06. 14704791 10.1038/nm982

[31] Goldberg SF, Miele ME, Hatta N, Takata M, Paquette-Straub C, Freedman LP, et al Melanoma metastasis suppression by chromosome 6: evidence for a pathway regulated by CRSP3 and TXNIP. Cancer Res. 2003;63(2):432–40. Epub 2003/01/25. 12543799

[32] Schmidt M, Bohm D, von Torne C, Steiner E, Puhl A, Pilch H, et al The humoral immune system has a key prognostic impact in node-negative breast cancer. Cancer Res. 2008;68(13):5405–13. Epub 2008/07/03. 10.1158/0008-5472.CAN-07-5206 18593943

[33] Teschendorff AE, Miremadi A, Pinder SE, Ellis IO, Caldas C. An immune response gene expression module identifies a good prognosis subtype in estrogen receptor negative breast cancer. Genome Biol. 2007;8(8):R157 Epub 2007/08/09. 17683518 10.1186/gb-2007-8-8-r157 PMC2374988

[34] Dai H, van't Veer L, Lamb J, He YD, Mao M, Fine BM, et al A cell proliferation signature is a marker of extremely poor outcome in a subpopulation of breast cancer patients. Cancer Res. 2005;65(10):4059–66. 15899795 10.1158/0008-5472.CAN-04-3953

[35] Leivonen SK, Kahari VM. Transforming growth factor-beta signaling in cancer invasion and metastasis. Int J Cancer. 2007;121(10):2119–24. Epub 2007/09/13. 17849476 10.1002/ijc.23113

[36] Qamri Z, Preet A, Nasser MW, Bass CE, Leone G, Barsky SH, et al Synthetic cannabinoid receptor agonists inhibit tumor growth and metastasis of breast cancer. Molecular cancer therapeutics. 2009;8(11):3117–29. Epub 2009/11/06. 10.1158/1535-7163.MCT-09-0448 19887554 PMC4128286

[37] Essayan DM. Cyclic nucleotide phosphodiesterases. The Journal of allergy and clinical immunology. 2001;108(5):671–80. Epub 2001/11/03. 11692087 10.1067/mai.2001.119555

[38] Yang H, Rouse J, Lukes L, Lancaster M, Veenstra T, Zhou M, et al Caffeine suppresses metastasis in a transgenic mouse model: a prototype molecule for prophylaxis of metastasis. Clin Exp Metastasis. 2005;21(8):719–35. 10.1007/s10585-004-8251-4 16035617

[39] Magenta G, Borenstein X, Rolando R, Jasnis MA. Rosiglitazone inhibits metastasis development of a murine mammary tumor cell line LMM3. BMC Cancer. 2008;8:47 10.1186/1471-2407-8-47 18261208 PMC2268944

[40] Singh A, Loscalzo J, Brigham and Women's Hospital. The Brigham intensive review of internal medicine. Second edition. ed. p. p.

[41] Crawford NP, Ziogas A, Peel DJ, Hess J, Anton-Culver H, Hunter KW. Germline polymorphisms in SIPA1 are associated with metastasis and other indicators of poor prognosis in breast cancer. Breast Cancer Res. 2006;8(2):R16 16563182 10.1186/bcr1389 PMC1483843

[42] Hsieh SM, Look MP, Sieuwerts AM, Foekens JA, Hunter KW. Distinct inherited metastasis susceptibility exists for different breast cancer subtypes: a prognosis study. Breast Cancer Res. 2009;11(5):R75 Epub 2009/10/15. 10.1186/bcr2412 19825179 PMC2790856

[43] Gaudet MM, Hunter K, Pharoah P, Dunning AM, Driver K, Lissowska J, et al Genetic variation in SIPA1 in relation to breast cancer risk and survival after breast cancer diagnosis. Int J Cancer. 2009;124(7):1716–20. Epub 2008/12/18. 10.1002/ijc.23919 19089925 PMC2914460

[44] Kim HJ, Kwon H, Lee JW, Kim HJ, Lee SB, Park HS, et al Metformin increases survival in hormone receptor-positive, HER2-positive breast cancer patients with diabetes. Breast Cancer Res. 2015;17(64). 10.1186/s13058-015-0574-3 PMC4504447 25935404

[45] El-Haggar SM, El-Shitany NA, Mostafa MF, El-Bassiouny NA. Metformin may protect nondiabetic breast cancer women from metastasis. Clin Exp Metastasis. 2016. Epub 2016 Feb 22. 10.1007/s10585-016-9782-1 26902691

[46] Bayraktar S, Hernadez-Aya LF, Lei X, Meric-Bernstam F, Litton JK, Hsu L, et al Effect of metformin on survival outcomes in diabetic patients with triple receptor-negative breast cancer. Cancer. 2012;118(5):1202–11. 10.1002/cncr.26439 21800293 PMC3207034

[47] He H, Ke R, Lin H, Ying Y, Liu D, Luo Z. Metformin, an old drug, brings a new era to cancer therapy. Cancer J. 2015;21(2):70–4. 10.1097/PPO.0000000000000103 25815846 PMC5588661

[48] Pei XF, Noble MS, Davoli MA, Rosfjord E, Tilli MT, Furth PA, et al Explant-cell culture of primary mammary tumors from MMTV-c-Myc transgenic mice. In Vitro Cell Dev Biol Anim. 2004;40(1–2):14–21. 15180438 10.1290/1543-706X(2004)40<14:ECOPMT>2.0.CO;2

