# Supplementary figures and images for: An Integrated Genome-Wide Systems Genetics Screen for Breast Cancer Metastasis Susceptibility Genes
Source: PLoS Genet. 2016 Apr 13;12(4):e1005989. doi: 10.1371/journal.pgen.1005989 (PMC4830524; doi:10.1371/journal.pgen.1005989)

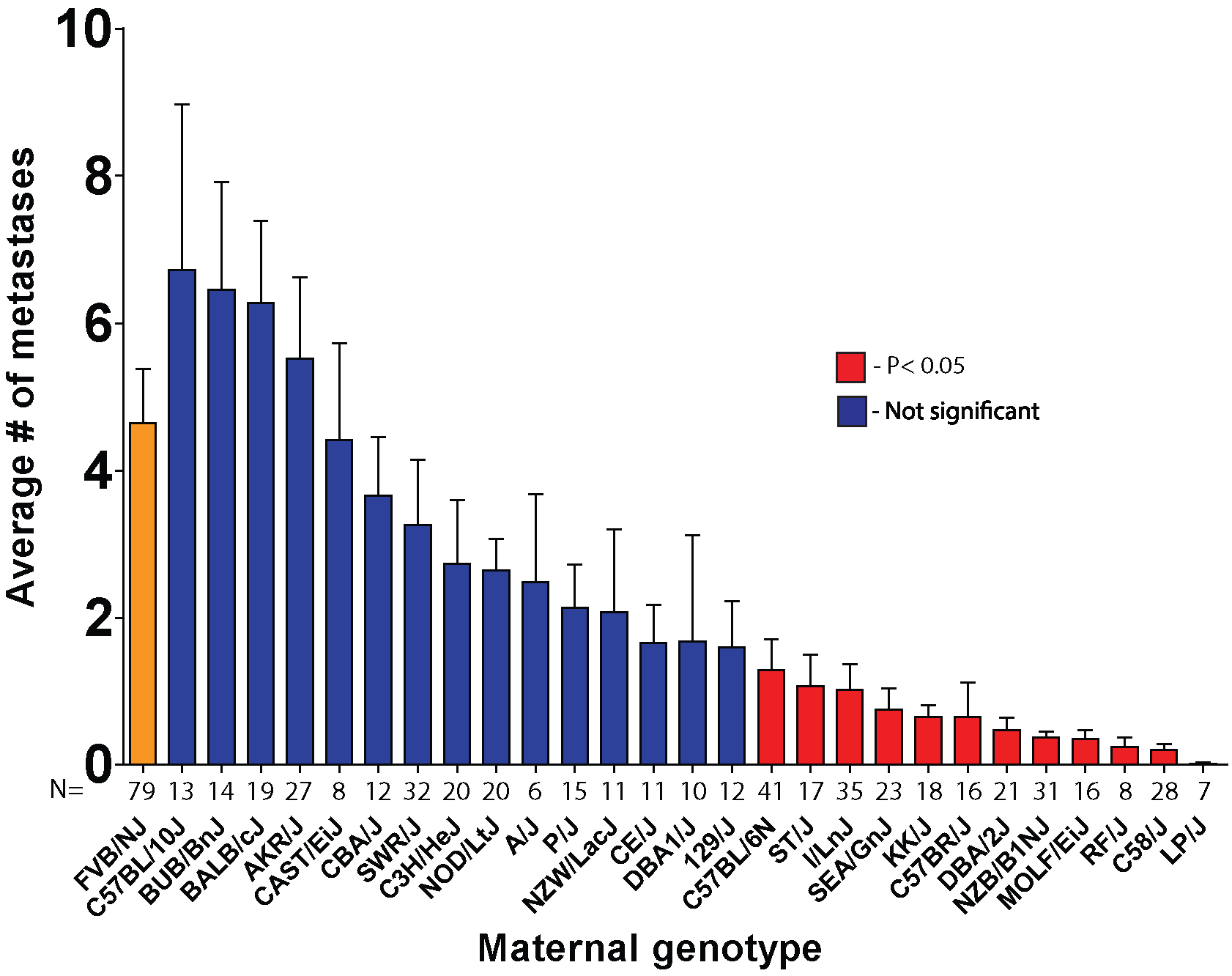

Supplement: S1 Fig — The original FVB/NJ MMTV-PyMT genetic background is indicated by the gold histogram bar. Strains not significantly different from MMTV-PyMT are indicated in blue. Stains that significantly suppress metastasis are indicated with red bars. The number of animals for each genotype is indicated under the X-axis. (TIF) [file pgen.1005989.s001.tif]

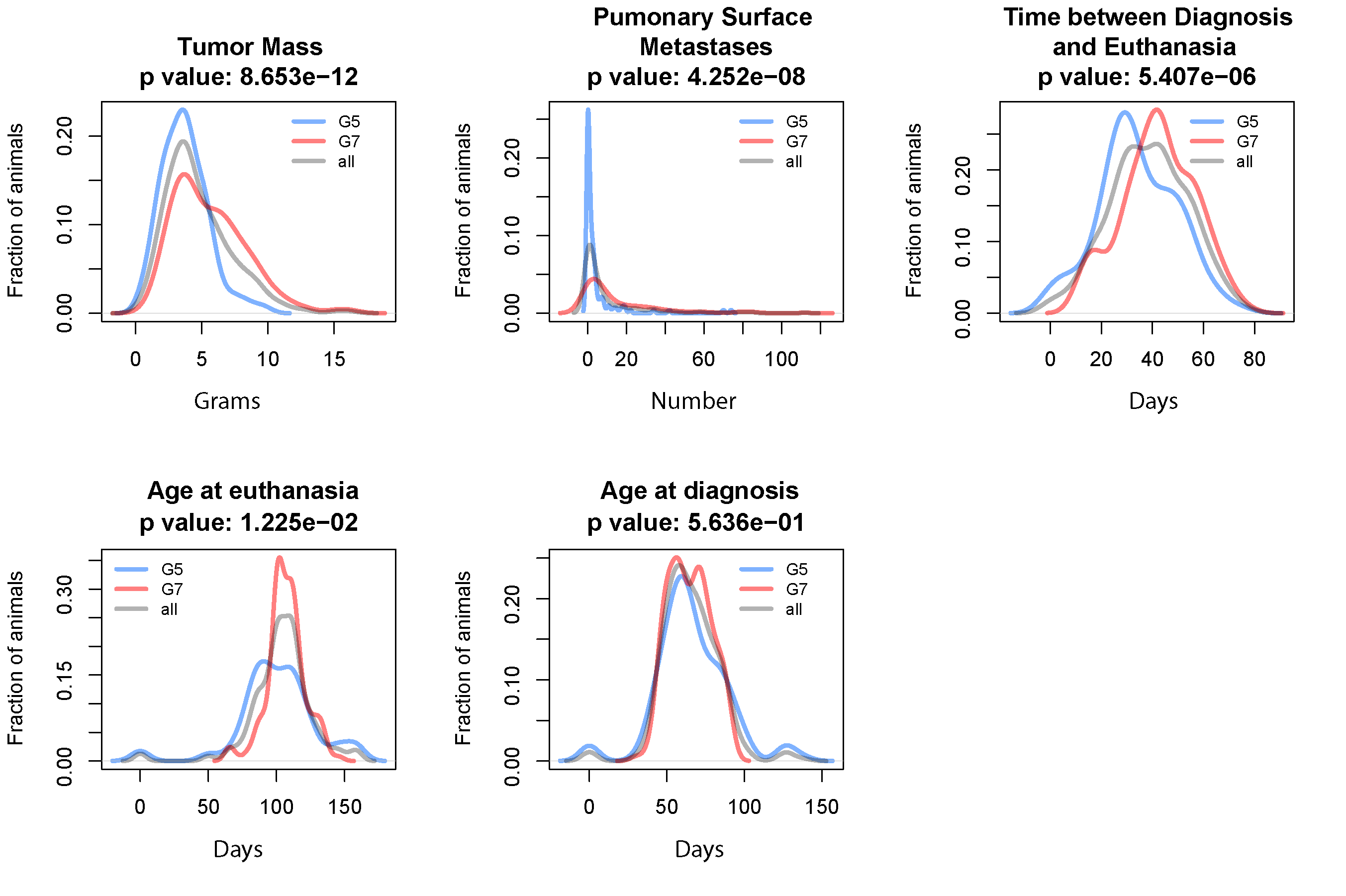

Supplement: S2 Fig — (TIF) [file pgen.1005989.s002.tif]

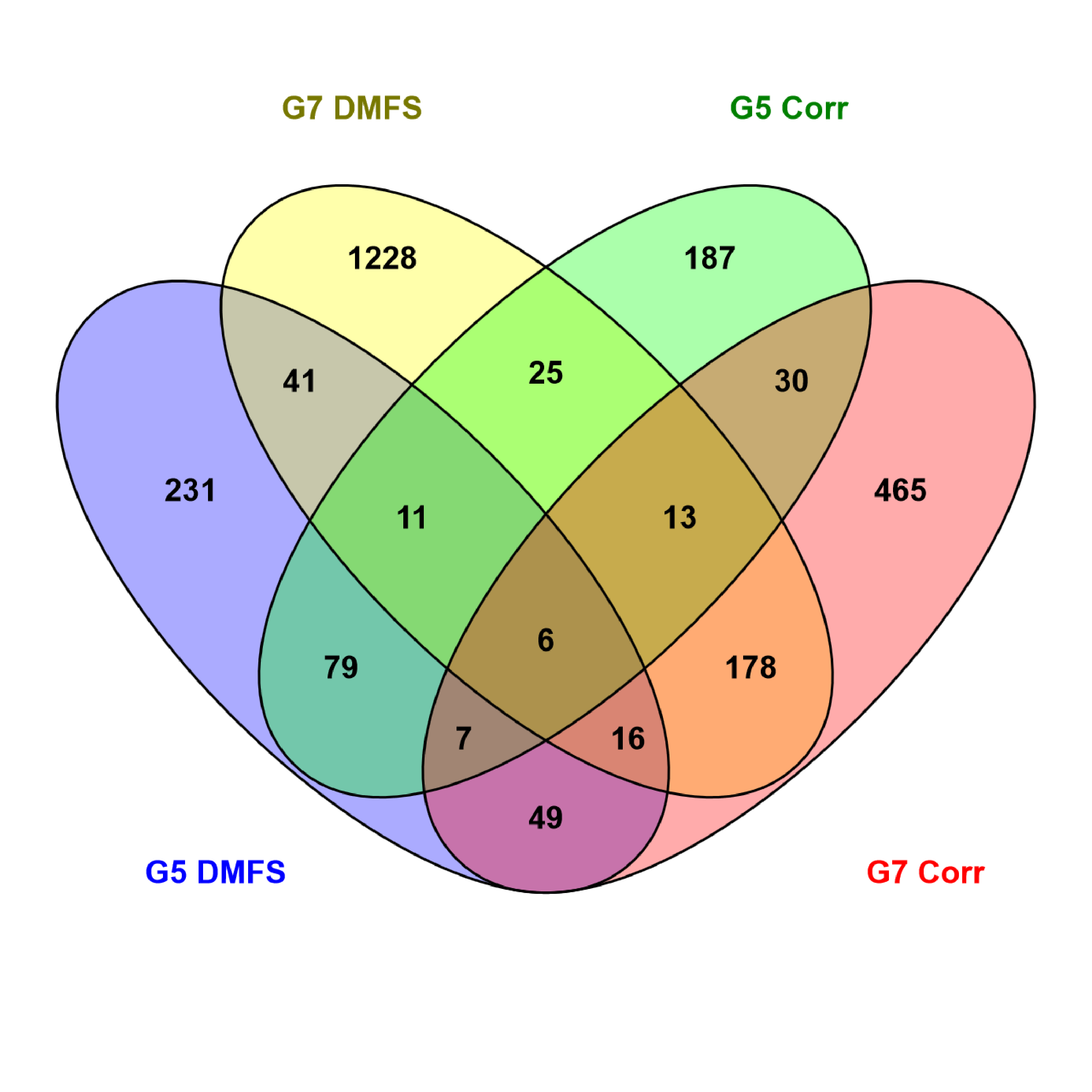

Supplement: S3 Fig — (TIF) [file pgen.1005989.s003.tif]

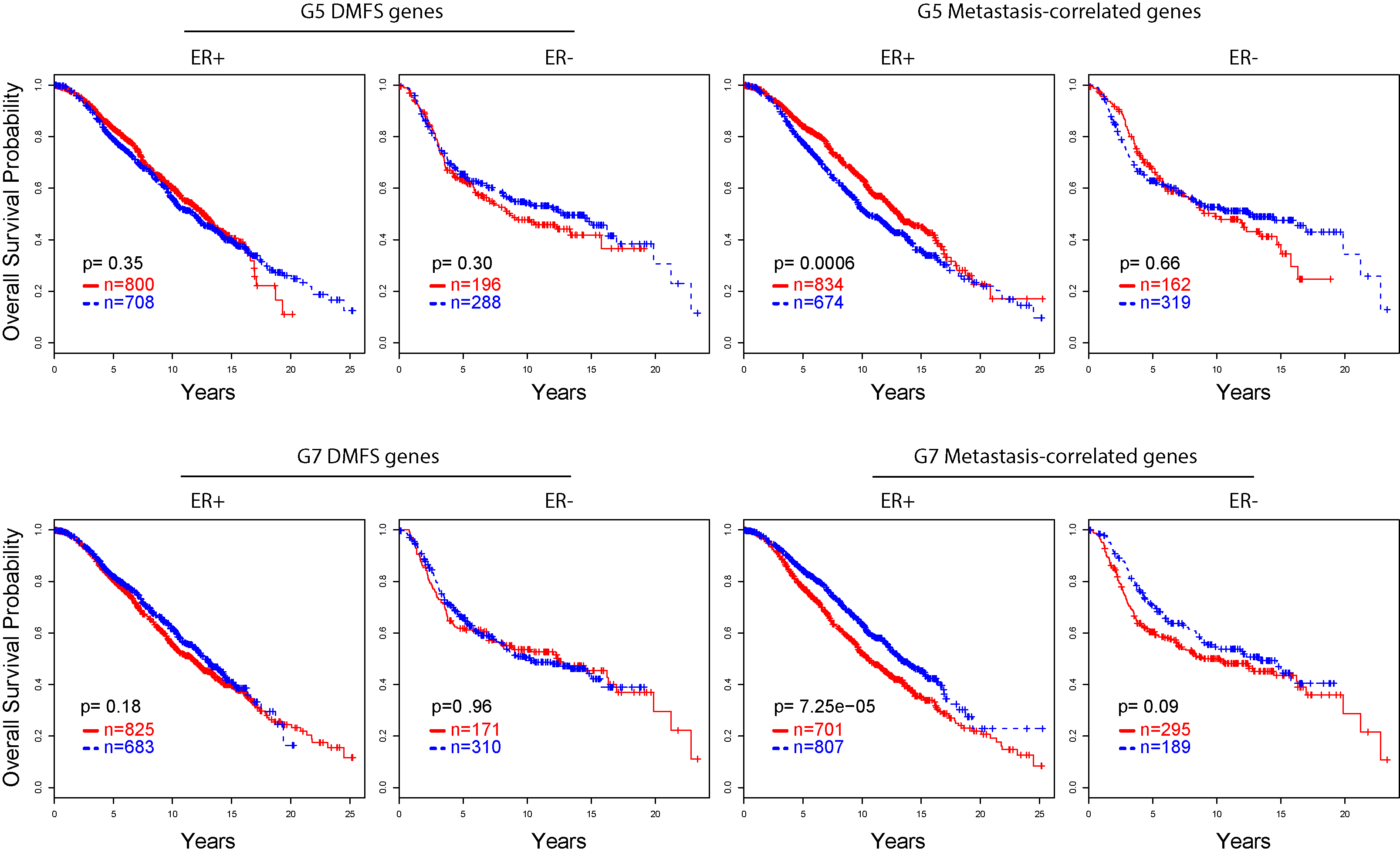

Supplement: S4 Fig — Each gene signature was tested separately on the estrogen receptor-positive (ER+) or estrogen receptor-negative (ER-) subsets of patient data sets. (TIF) [file pgen.1005989.s004.tif]

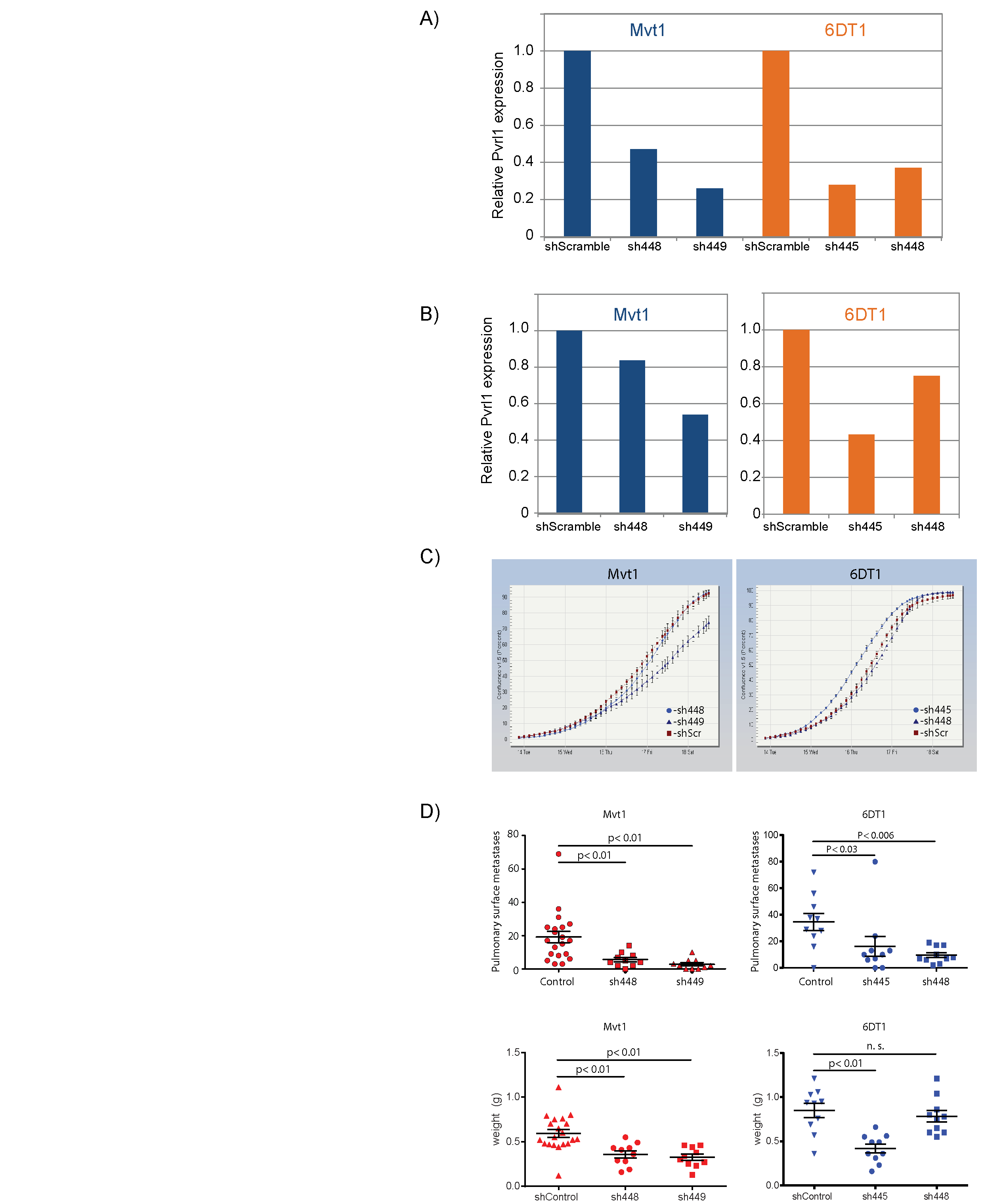

Supplement: S5 Fig — A) In vitro qRT-PCR analysis of shRNA knockdowns of Pvrl1 in Mvt1 and 6DT1 cells showing the relative expression in the knockdown cells compared to shScramble controls. B) In vivo qRT-PCR analysis of shRNA knockdowns of Pvrl1 in Mvt1 and 6DT1 implanted tumors. N = 5 for each group. C) In vitro proliferation assays for the shRNA knockdowns in Mvt1 and 6DT1 cells as measured on the Incucyte ZOOM instrument. D) Pulmonary surface metastases and orthotopic tumor weight results for mammary fat pad implantation of Pvrl1 shRNA knockdown Mvt1 and 6DT1 cells. P values represent the result of an ANOVA test after Dunnetts correction for multiple comparisons against the shControl data. (TIF) [file pgen.1005989.s005.tif]

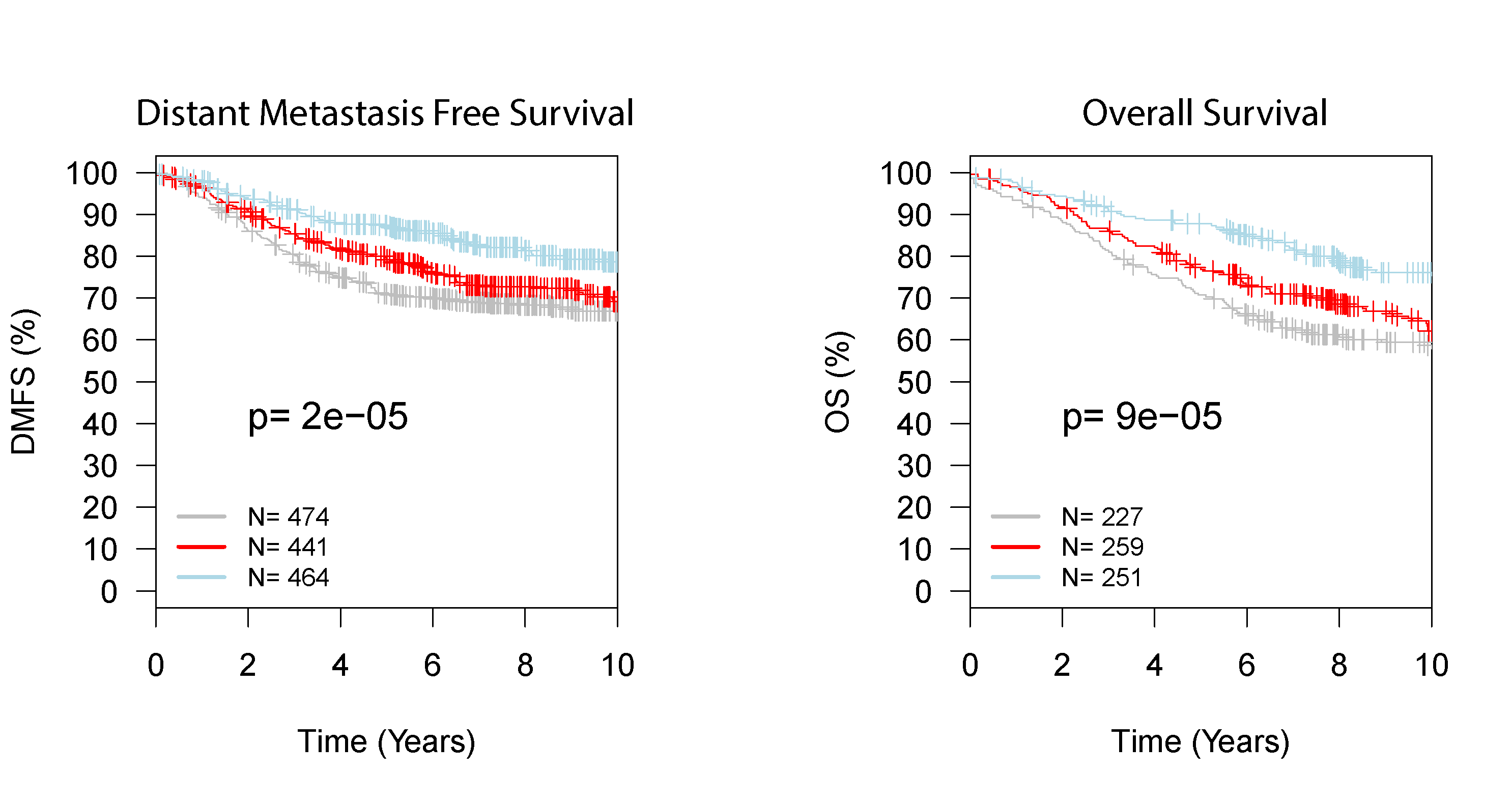

Supplement: S7 Fig — (TIF) [file pgen.1005989.s007.tif]
